# Recognition of worsening heart failure symptoms, help-seeking attitudes, and time-to-presentation in chronic heart failure: a single-center KAP study in China

**DOI:** 10.3389/fcvm.2026.1713344

**Published:** 2026-03-13

**Authors:** Han Han

**Affiliations:** Cardiology, The Affiliated Hospital of Xuzhou Medical University, Xuzhou, Jiangsu, China

**Keywords:** China, heart failure, help-seeking, self-care, symptom recognition, time-to-presentation

## Abstract

**Background:**

Timely recognition and prompt help-seeking for worsening heart failure (HF) mitigate deterioration, yet patient-side delays remain common in China. This study evaluated how knowledge, attitudes, and practices (KAP) relate to time-to-presentation (TTP).

**Methods:**

In a single-center cross-sectional study (March 2022–April 2025), adults with chronic HF completed a standardized KAP instrument; clinical data were abstracted. Among those with a worsening episode in the prior six months, TTP was categorized (≤6, 7–12, 13–24, 25–48, >48 h). Prespecified analyses comprised logistic regression for early presentation (≤6 h) adjusted for education, NYHA class, knowledge (per-point), written action plan, and insurance; ordinal models for knowledge categories; and structural-equation modeling of the Knowledge→Attitudes→Self-care→TTP pathway.

**Results:**

Among the 2,110 patients, recognition was highest for breathlessness/edema/fatigue (80%–85%), ∼60% for weight gain/orthopnea, and lowest for paroxysmal nocturnal dyspnea, dizziness/fainting, confusion/sleepiness, and oliguria (45%, 42%, 39%, 33%). Attitudes were favorable (early help-seeking 90%; trust 87%; self-efficacy 78%), but barriers were common (clinic hours/crowding 42%; costs 37%; transport 29%). Self-care diverged (medication adherence 83%; daily weight 21%). Latent classes (Proactive, Ambivalent, Barrier-Focused) and knowledge domains (Classical, Advanced, Acute-change) were observed. Among those with recent worsening (*n* = 1,319), early presentation was more common with university + education (47.7% vs. 35.2%), higher NYHA (IV 48.9%, III 47.2%, I–II 39.1%), and high knowledge (50.5% vs. 31.9%; *p* < 0.001). Independent predictors of early presentation were NYHA IV (aOR 2.01, 95% CI 1.32–3.06), NYHA III (1.52, 1.17–1.98), and knowledge (per point 1.14, 1.07–1.22); lower education (0.68, 0.49–0.94), no action plan (0.71, 0.54–0.93), and no insurance (0.59, 0.37–0.94) reduced odds. Structural modelling indicated a knowledge effect (β = 0.423) mediated by attitudes and self-care.

**Conclusion:**

Content-specific education, written action plans, and access facilitation—particularly for lower-education and uninsured groups—are pragmatic levers to shorten patient-dependent delays in worsening HF.

## 1 Introduction

Heart failure (HF) remains a leading cause of morbidity, mortality, and expenditure worldwide, with a rising burden despite therapeutic advances (1, 2). In China, population ageing and high cardiometabolic risk have sustained increases in HF prevalence and disability, underscoring the need for prevention and timely escalation of care along the chronic continuum (3, 4). Guidelines emphasize that early recognition of decompensation and prompt help-seeking avert deterioration, hospitalization, and death, particularly for congestion-dominant “worsening” episodes (4). Yet patients’ capacity to appraise symptom change and act decisively varies, shaped by knowledge, attitudes, self-efficacy, and access—domains amenable to public-health intervention (1, 5–8).

Symptom perception and self-care sit at the core of effective HF management. The situation-specific theory posits that maintenance behaviors (e.g., weight monitoring, adherence) and management behaviors (recognizing, interpreting, responding to change) are influenced by person and context, with confidence mediating effects on outcomes (6, 9, 10). Psychometric work with the revised Self-Care of Heart Failure Index (SCHFI v7.2) supports these constructs and links better self-care to improved patient-centered outcomes (9, 11). In parallel, the “time-to-treatment” paradigm suggests that shorter intervals to intravenous diuretics in acute HF are associated—though not uniformly across settings—with better short-term outcomes, implicating both patient- and system-side delays as clinically consequential (7, 12–14). Accordingly, quantifying patients' knowledge of worsening signs, help-seeking attitudes, and time-to-presentation (TTP) is foundational for targeting interventions that shift behavior into the earliest therapeutic window.

Evidence connecting delays to adverse outcomes has strengthened. A prospective cohort showed that longer care-seeking delay after symptom onset predicted higher rehospitalization and mortality post-discharge (7, 9, 11). International registry and synthesis studies further associate prolonged time to diuretics with higher 30-day mortality, especially among high-risk patients (14, 15). Collectively, these data frame patient-side delay—not merely pre-hospital processes—as a modifiable determinant of outcomes.

China is a particularly important, yet incompletely characterized, setting for this behavioral pathway. National reports catalogue substantial cardiovascular burden and uneven capacity across urban and rural regions but offer limited granularity on behavioral precursors of timeliness in HF care (2, 14). Emerging domestic studies document gaps in the appraisal of dyspnea, orthopnea, oedema, weight change, and neurocognitive/renal signals and link poorer perception to worse self-care and quality of life (11, 16). Mixed-methods work further implicates symptom burden, depressive symptoms, pandemic-related constraints, caregiver support, and perceived system responsiveness in care-seeking delay among Chinese patients with HF (12, 69). Nevertheless, three interlocking gaps persist. First, Chinese KAP investigations remain recent and often narrow (e.g., home-based rehabilitation) or caregiver-focused, limiting inference about patient-level KAP constructs that activate timely help-seeking in worsening HF (17, 18). Second, few studies explicitly link KAP domains to clinically meaningful behavioral endpoints—categorical TTP following a worsening episode—leaving the translational bridge from knowledge and attitudes to observed timeliness under-quantified despite persuasive international evidence (9, 15, 19). Third, psychometric dissection of knowledge into clinically coherent domains—classical congestion signs, advanced/atypical features (e.g., oliguria, confusion), and rapid acute changes (e.g., abrupt weight gain, paroxysmal nocturnal dyspnea)—has not been integrated with help-seeking attitudes and real-world TTP in a single Chinese cohort, limiting the capacity to prioritize education content, tailor behavioral interventions, and redesign services toward the most behaviorally potent knowledge gaps (3, 7, 20).

Guideline frameworks and implementation reviews consistently foreground symptom recognition and rapid action plans, particularly for patients with recurrent decompensation (1, 6, 20). From a health-services standpoint, earlier presentation offers prospects of reduced congestion-related organ dysfunction and shorter length of stay, motivating scalable, behavior-focused interventions (7, 8, 13). Yet routine counselling in many Chinese clinics appears to privilege medication and diet over structured teaching on weight trajectories, nocturnal symptoms, oliguria, and cognitive change—signals frequently under-recognized but potentially decisive for early care (13, 17, 18). Economic and organizational barriers (e.g., perceived cost, clinic hours/crowding) may further dampen otherwise favorable help-seeking attitudes, especially in suburban and rural catchments with variable capacity (2, 12, 21). These realities argue for KAP-oriented surveillance embedded in clinical workflows to identify and modify antecedents of delay (1, 7, 8, 12, 17, 18, 22).

This single-center study addresses these gaps by concurrently measuring recognition of worsening HF symptoms, help-seeking attitudes, perceived access barriers, and categorical TTP following a recent worsening episode. Knowledge items were structured into clinically meaningful domains and evaluated psychometrically to align educational content with pathophysiology. Attitudinal heterogeneity was profiled by latent class analysis to identify segments for tailored intervention. Finally, multivariable and pathway models linked KAP constructs to TTP. The objective was to quantify domain-specific knowledge, characterize attitude profiles, and estimate adjusted associations between KAP, NYHA class, and early presentation, thereby identifying pragmatic levers for public-health action in China.

## 2 Methods

### 2.1 Study design and setting

This single-center, cross-sectional knowledge–attitudes–practices (KAP) investigation was conducted at Department of Cardiology, The affiliated Hospital of Xuzhou Medical University, Xuzhou Jiangsu, China, within routine ambulatory heart failure services from March 2022 through April 2025. The methodological objective was to quantify patients' recognition of worsening heart failure symptoms, delineate help-seeking orientations and access constraints, and relate these constructs to the timeliness of presentation after recent deterioration. Study procedures were standardized *a priori* to maximize measurement fidelity while maintaining external generalizability to the hospital's catchment population. The sampling strategy was pragmatic and consecutive; the available clinic volume over the study window was expected to yield a cohort sufficient to estimate adjusted associations of moderate magnitude with acceptable precision.

### 2.2 Participants and eligibility

Consecutive adults presenting for heart failure care were screened by trained research staff during scheduled encounters. Eligibility required age ≥18 years, a clinician-documented diagnosis of chronic heart failure [either heart failure with reduced ejection fraction (HFrEF) or heart failure with preserved ejection fraction (HFpEF)], and the capacity to complete an interviewer-administered survey unaided or with standard supports. Exclusion criteria were predefined to minimize measurement bias and participant burden and included hemodynamic instability or active decompensation that precluded interview at the visit; severe cognitive impairment, delirium, or advanced communication difficulties without an available proxy; inability to communicate in the study languages despite interpreter support; concurrent enrolment in another behavioral or educational study targeting symptom recognition or help-seeking; and any circumstance judged by the treating clinician to compromise reliable self-report (e.g., acute intoxication or severe psychiatric crisis). To characterize potential selection bias, screening logs summarized numbers approached, eligible, and enrolled, and the catchment area was described in terms of referral pathways and routine access patterns; generalizability is discussed with attention to patients who avoid routine care or present exclusively to emergency services. The analytic cohort comprised 2,110 participants with complete KAP data. A prespecified behavioral sub cohort—those reporting at least one worsening heart failure episode within the preceding six months—formed the denominator for timeliness analyses (*n* = 1,319).

### 2.3 Data sources and collection procedures

Data were obtained contemporaneously through a structured, interviewer-administered instrument and parallel chart abstraction conducted under written operating procedures. The instrument captured sociodemographic; item-level knowledge of worsening heart failure signs; help-seeking attitudes; perceived barriers to accessing care; self-care practices and the presence of a written, clinician-endorsed action plan; and, for those with a recent episode, the self-reported interval from symptom onset to first medical contact. Chart abstraction supplied heart failure phenotype, New York Heart Association (NYHA) functional class, years since diagnosis, health-care utilization (visits/year), and heart failure hospitalizations over the previous 12 months. Interviewers received multisession central training with competency checks and scripted prompts; range and logic checks were embedded to reduce interviewer and respondent error; and a random subset of charts underwent duplicate abstraction with adjudication of discrepancies. To mitigate recall bias for TTP, interviewers used neutral day–night and weekday–weekend anchors, and when feasible, self-reported intervals were cross-checked against electronic timestamps (e.g., triage or registration times) in a verification subset. Planned future work considers a shorter recall window to further limit recall error.

### 2.4 Variables and measurements

Sociodemographic variables included age (years, continuous and categorized as <50, 50–64, 65–74, ≥75), sex (male, female), highest educational attainment (no formal education, primary school, secondary school, college, university, postgraduate), primary insurance coverage (public, private, mixed, none), living arrangement (lives alone, lives with spouse/partner, lives with extended family, institutional/other), and usual residence (urban, suburban, rural). Clinical variables comprised heart failure phenotype (HFrEF, HFpEF, unknown), the most recent clinician-assigned NYHA functional class (I–IV, with an explicit unknown/not documented level retained for descriptive completeness), the number of heart failure–related hospitalizations in the prior 12 months (none, 1, 2, 3, ≥4), years since first documented heart failure diagnosis (continuous), and annual health-care utilization (cardiovascular visits/year, continuous).

Knowledge of worsening heart failure was measured with a 12-item bank aligned to guideline-concordant warning signs. For each item—breathlessness at rest; lower-extremity oedema; exercise intolerance/fatigue; orthopnea; paroxysmal nocturnal dyspnea; nocturnal cough/wheeze; abdominal swelling/early satiety; palpitations/fast heartbeat; dizziness/fainting; new confusion/excessive sleepiness; oliguria; and rapid weight gain (>2 kg in 3 days)—respondents indicated Yes/No/Unsure as to whether the sign signaled worsening heart failure. Responses were scored 1 for correct recognition and 0 for incorrect or unsure, yielding a total knowledge score from 0 to 12; for some analyses, scores were categorized as Low (0–4), Moderate (5–8), and High (9–12). Administratively, interviewers read each item verbatim and used standardized neutral prompts where clarification was needed, instructing respondents to judge each sign independently even when symptoms co-occurred. Psychometric evaluation prespecified principal-axis EFA with oblique (oblimin) rotation on nine core warning signs identified during pilot testing for acceptable item–test correlations; three lower-performing items (nocturnal cough/wheeze, abdominal swelling/early satiety, palpitations) were retained in the 12-item clinical knowledge score but excluded from EFA on empirical grounds. Sampling adequacy was excellent (KMO = 0.847). Factor retention used parallel analysis and the minimum average partial criterion. Items were assigned by primary rotated loading ≥0.40 with cross-loadings ≥0.30 flagged; communalities (*h*^2^) are reported in Table 7. Internal consistency was summarized by Cronbach's α for each domain. Content validity was established through review by a multidisciplinary expert panel (cardiology, nursing, emergency medicine, behavioral science), and cultural/linguistic adaptation followed forward-translation, back-translation, and cognitive interviewing with patients to ensure conceptual equivalence. Criterion-related validity was explored by examining associations between knowledge metrics and behavioral endpoints in the inferential models.

Help-seeking attitudes were captured with five Likert-scaled statements (Strongly agree to Strongly disagree) addressing perceived efficacy of early help-seeking, trust in the local clinic/emergency department, confidence in arranging medical help (self-efficacy), and two barriers—medical expenses and embarrassment—as potential deterrents. For descriptive summaries, agreement was defined as Strongly agree or Agree. To characterize attitudinal heterogeneity, these domains were entered into latent class analysis; competing models were compared on information criteria, and a three-class solution—Proactive, Ambivalent, and Barrier-focused—was retained based on parsimony and interpretability, with classification accuracy summarized by domain and residual associations inspected for local independence.

Perceived access barriers and self-care practices were measured using ordered response categories. Barriers (cost of medical care, transportation difficulties, clinic hours/crowding) were rated Not at all, Minor, Moderate, or Major; a derived Moderate + Major indicator summarized salient burden for each barrier. Behaviors were captured for daily weight monitoring and medication adherence using a five-point frequency scale (Never, Rarely, Sometimes, Often, Always); good adherence was defined *a priori* as Often or Always. The presence of a written or clinician-endorsed action plan specified symptom thresholds and steps for seeking care and was recorded as Yes/No.

Worsening heart failure episodes were ascertained by asking participants whether they had experienced a symptomatic deterioration consistent with heart failure within the prior six months. Episodes were patient-reported and were not independently adjudicated by a clinician; accordingly, some episodes may have reflected non–heart failure etiologies (e.g., anaemia, respiratory illness) that produce overlapping symptoms. Among those responding “Yes”, TTP was elicited as the interval from symptom onset to first medical contact and recorded in ordered categories (≤6 h, 7–12 h, 13–24 h, 25–48 h, >48 h), with a cannot recall option preserved to avoid forced classification. The elapsed time between the worsening episode and the study interview was not systematically recorded; therefore, recall intervals varied across participants, and longer intervals may have introduced greater imprecision in self-reported TTP.

### 2.5 Data quality assurance and statistical analysis

Quality safeguards comprised trained interviewers, piloted administration, embedded range/logic checks, random duplicate chart abstraction with adjudication, and audited data management. Continuous variables were summarized as means (standard deviations) and categorical variables as counts (percentages); group differences in TTP across education, NYHA class, and knowledge used Pearson's *χ*^2^ (two-sided α = 0.05). The primary model was a multivariable logistic regression of early presentation (≤6 h) in the recent-episode subcohort, adjusted for prespecified covariates—age (continuous, per 10-year increase), sex (female vs. male), education (No formal/Primary vs. University+), NYHA class (III and IV vs. I–II), knowledge score (per 1-point increase), presence of a written action plan (No vs. Has plan), and insurance (No insurance vs. Public)—with adjusted odds ratios and 95% confidence intervals reported. Age and sex were included *a priori* as core confounders given their established associations with cardiovascular help-seeking behavior. Attitudinal heterogeneity was characterized by latent class analysis (three-class solution selected by AIC, BIC, sample-size adjusted BIC, entropy, and BLRT), and a structural equation model specified Knowledge → Positive attitudes → Self-care behaviors → Early presentation plus the total effect of NYHA class, estimated with a robust categorical estimator (WLSMV). Analyses used R (psych, ordinal, mice, lavaan) with poLCA for latent classes and Stata for verification. Missing data were handled under a prespecified framework: patterns/percentages summarized; mechanisms assessed by Little's MCAR test and targeted models of missingness; primary analyses complete case; sensitivity analyses used multiple imputation by chained equations (predictive mean matching for continuous variables; ordinal/polytomous models for categorical variables) with adequate imputations, convergence diagnostics, and stability checks; participants answering *cannot recall* for TTP were excluded from the primary behavioral model and included in ordinal sensitivity analyses. Coding was prespecified: education as a six-level ordered factor with a primary contrast of No formal/Primary vs. University+, and NYHA unknown/not documented excluded in primary models but retained as a category in robustness checks; model performance for the early-presentation regression was reported by AUC (95% CI) and calibration (Hosmer–Lemeshow-type), with bootstrap optimism correction.

## 3 Results

### 3.1 Participant characteristics

This cross-sectional study enrolled 2,110 adults with chronic heart failure. The mean age was 65.8 ± 12.4 years (range 24–93); 54.3% were male (*n* = 1,146) and 45.7% female (*n* = 964). The age distribution comprised <50 years (12.0%), 50–64 years (28.0%), 65–74 years (38.0%), and ≥75 years (22.0%). Regarding living arrangement, 14.0% lived alone, 38.0% lived with a spouse or partner, 44.0% lived with extended family, and 4.0% were in institutional or other settings. Educational attainment showed considerable diversity, with primary school representing the largest group (30.0%), followed by secondary school (23.0%), college (20.0%), university (17.0%), no formal education (6.0%), and postgraduate degrees (4.0%). Most participants relied on public insurance coverage (70.0%), while 12.0% had private insurance, 9.0% had mixed coverage, and 9.0% lacked any insurance. Residential distribution was fairly balanced across urban (40.0%), suburban (35.0%), and rural (25.0%) settings. Heart failure phenotypes included HFrEF in half the cohort (50.0%), HFpEF in 40.0%, and unknown classification in 10.0%. NYHA functional class distribution showed Class II as predominant (50.0%), followed by Class III (30.0%), Class I (10.0%), and Class IV (8.0%), with 2.0% having unknown or undocumented status. Most participants experienced no heart failure hospitalizations in the preceding 12 months (60.0%), while 26.0% had one hospitalization, 9.0% had two, 3.0% had three, and 2.0% had four or more admissions. The knowledge score averaged 7.2 ± 2.8 on the 0–12 scale with a full range of scores observed. A substantial proportion of participants (62.5%, *n* = 1,319) reported experiencing a worsening heart failure episode within the past six months, forming the analytical cohort for TTP analyses (Table 1).

**Table 1:** Participant demographics and clinical characteristics (*N* = 2,110).

Characteristic	No. (%)
Age, years	
Mean ± SD	65.8 ± 12.4
Range	24–93
<50 years	253 (12.0)
50–64 years	591 (28.0)
65–74 years	802 (38.0)
≥75 years	464 (22.0)
Sex	
Male	1,146 (54.3)
Female	964 (45.7)
Living arrangement	
Lives alone	295 (14.0)
Lives with spouse/partner	802 (38.0)
Lives with extended family	928 (44.0)
Institutional/other	85 (4.0)
Education level	
No formal education	127 (6.0)
Primary school	634 (30.0)
Secondary school	486 (23.0)
College	422 (20.0)
University	358 (17.0)
Postgraduate	83 (4.0)
Insurance type	
Public	1,476 (70.0)
Private	253 (12.0)
Mixed	190 (9.0)
None	191 (9.0)
Residence	
Urban	844 (40.0)
Suburban	739 (35.0)
Rural	527 (25.0)
Heart failure type	
HFrEF	1,055 (50.0)
HFpEF	844 (40.0)
Unknown	211 (10.0)
NYHA functional class	
Class I	211 (10.0)
Class II	1,055 (50.0)
Class III	633 (30.0)
Class IV	169 (8.0)
Unknown/Not documented	42 (2.0)
HF hospitalizations in past 12 months	
None	1,266 (60.0)
1	548 (26.0)
2	190 (9.0)
3	63 (3.0)
≥4	43 (2.0)
Knowledge score (0–12 scale)	
Mean ± SD	7.2 ± 2.8
Range	0–12
Worsening HF episode in past 6 months	
Yes	1,319 (62.5)
No	791 (37.5)

HF, heart failure; HFpEF, heart failure with preserved ejection fraction; HFrEF, heart failure with reduced ejection fraction; NYHA, New York heart association; SD, standard deviation.

### 3.2 Knowledge of heart failure warning signs

Recognition rates for heart failure warning signs demonstrated substantial variation across symptom domains. Classical signs of decompensation achieved high recognition rates, with breathlessness at rest recognized by 85.0% of participants, lower extremity edema by 82.9%, and exercise intolerance or fatigue by 80.0%. Intermediate recognition occurred for rapid weight gain exceeding 2 kg in 3 days (60.0%) and orthopnea or difficulty breathing when lying flat (58.0%). Additional symptoms showed moderate recognition patterns, including abdominal swelling or early satiety (55.0%), nocturnal cough or wheeze (52.0%), and palpitations or fast heartbeat (48.0%). The most challenging warning signs to recognize included paroxysmal nocturnal dyspnea (45.0%), dizziness or fainting (42.0%), new confusion or excessive sleepiness (39.0%), and oliguria or decreased urination (33.0%). Across all knowledge items, uncertainty responses ranged from 7.0% to 22.0%, while incorrect responses varied from 8.0% to 46.0%, suggesting considerable knowledge gaps in the more advanced or subtle manifestations of heart failure deterioration (Table 2).

**Table 2:** Knowledge of heart failure warning signs (*N* = 2,110).

Symptom/Sign	Correct recognition	Incorrect recognition	Uncertain
	(“Yes”)	(“No”)	(“Unsure”)
	No. (%)	No. (%)	No. (%)
Breathlessness at rest	1,793 (85.0)	169 (8.0)	148 (7.0)
Lower extremity edema	1,750 (82.9)	190 (9.0)	170 (8.1)
Exercise intolerance/fatigue	1,687 (80.0)	232 (11.0)	191 (9.0)
Rapid weight gain (>2 kg in 3 days)	1,266 (60.0)	422 (20.0)	422 (20.0)
Orthopnea (difficulty breathing when lying flat)	1,224 (58.0)	465 (22.0)	421 (20.0)
Abdominal swelling/early satiety	1,160 (55.0)	507 (24.0)	443 (21.0)
Nocturnal cough/wheeze	1,097 (52.0)	549 (26.0)	464 (22.0)
Palpitations/fast heartbeat	1,013 (48.0)	633 (30.0)	464 (22.0)
Paroxysmal nocturnal dyspnea	950 (45.0)	739 (35.0)	421 (20.0)
Dizziness/fainting	886 (42.0)	780 (37.0)	444 (21.0)
New confusion/excessive sleepiness	823 (39.0)	822 (39.0)	465 (22.0)
Oliguria (decreased urination)	697 (33.0)	971 (46.0)	442 (21.0)

### 3.3 Help-seeking attitudes and healthcare barriers

Participants demonstrated generally favorable attitudes toward help-seeking for worsening heart failure symptoms. Strong consensus emerged regarding the efficacy of early medical intervention, with 90.0% expressing agreement that early help-seeking prevents heart failure complications (60.0% strongly agreed, 30.0% agreed). Trust in local healthcare facilities was also high, with 87.0% reporting confidence in their local clinic or emergency department (40.0% strongly agreed, 47.0% agreed). Self-efficacy in arranging medical help showed moderate endorsement, with 78.0% expressing confidence in their ability to obtain care (28.0% strongly agreed, 50.0% agreed). However, financial constraints represented a significant concern, with 43.0% of participants agreeing that medical expenses prevent them from seeking help (17.0% strongly agreed, 26.0% agreed). Embarrassment emerged as a less prevalent but notable barrier, with 19.0% acknowledging that embarrassment prevents help-seeking (6.0% strongly agreed, 13.0% agreed) (Table 3).

**Table 3:** Attitudes toward help-seeking for worsening heart failure (*N* = 2,110).

Attitude Statement	Strongly	Agree	Neutral	Disagree	Strongly	Agreement^a^
	Agree	No. (%)	No. (%)	No. (%)	Disagree	No. (%)
	No. (%)				No. (%)	
Early medical help-seeking prevents HF complications	1,266 (60.0)	633 (30.0)	148 (7.0)	42 (2.0)	21 (1.0)	1,899 (90.0)
I trust my local clinic/emergency department	844 (40.0)	991 (47.0)	190 (9.0)	63 (3.0)	22 (1.0)	1,835 (87.0)
I am confident in arranging medical help	591 (28.0)	1,055 (50.0)	338 (16.0)	105 (5.0)	21 (1.0)	1,646 (78.0)
Medical expenses prevent me from seeking help	358 (17.0)	549 (26.0)	486 (23.0)	507 (24.0)	210 (10.0)	907 (43.0)
Embarrassment prevents me from seeking help	127 (6.0)	274 (13.0)	549 (26.0)	780 (37.0)	380 (18.0)	401 (19.0)

^a^ Agreement = Combined “Strongly Agree” and “Agree” responses.

**Figure 1:**
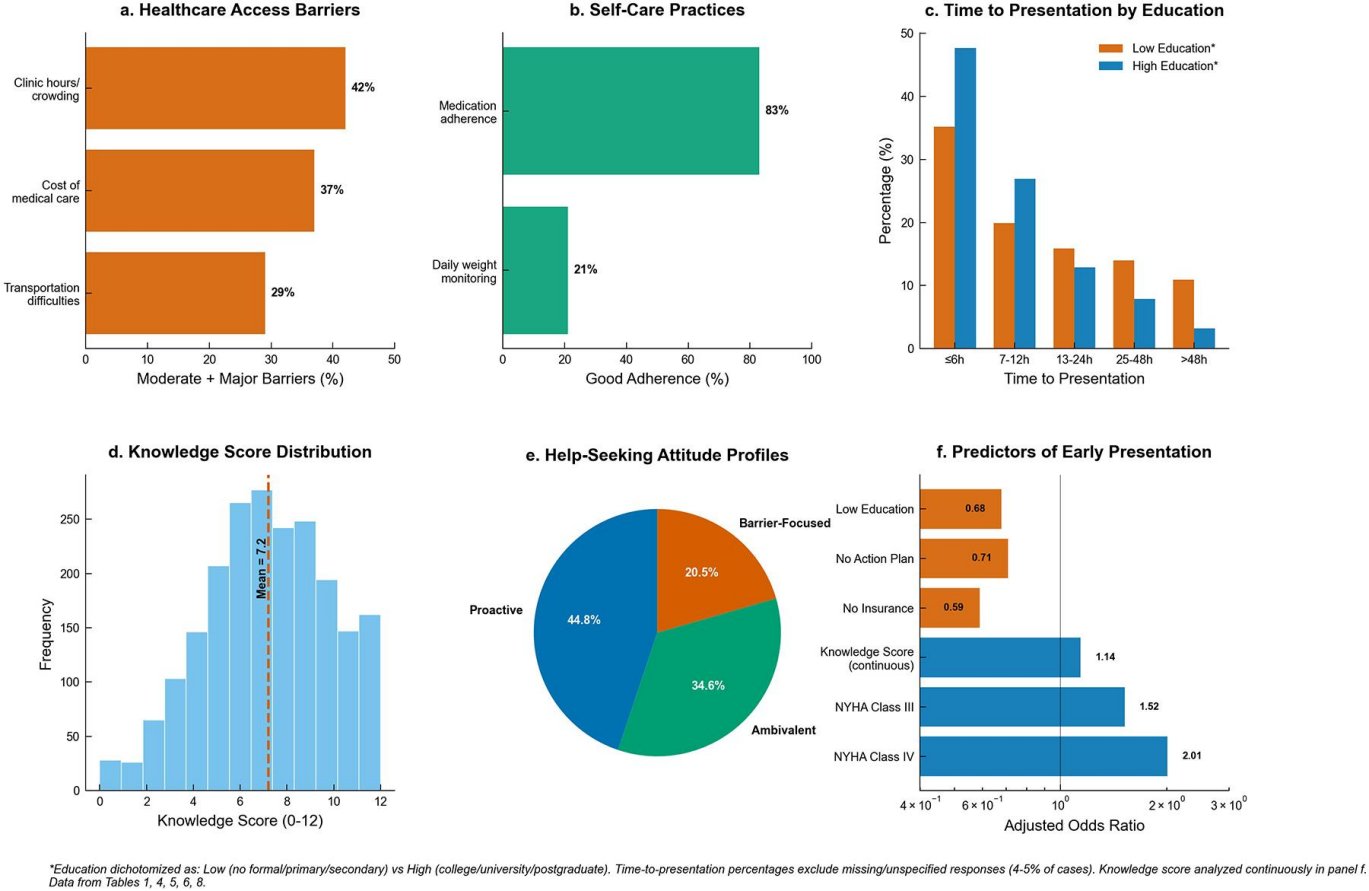
Heart failure knowledge, attitudes, and practices (*N* = 2,110). **(a)** Healthcare access barriers showing moderate and major burden percentages. **(b)** Self-care practices showing good adherence rates (often/always). **(c)** Time-to-presentation by education level among participants with recent worsening episodes, with percentages excluding missing responses (4%–5% of cases). **(d)** Knowledge score distribution with mean (7.2) and standard deviation (2.8). **(e)** Help-seeking attitude profiles from latent class analysis. **(f)** Forest plot showing adjusted odds ratios for early presentation (≤6 h) with 95% confidence intervals. Education contrast uses No formal/Primary vs. University+; percentages exclude missing/unspecified responses. Knowledge score analyzed continuously.

Healthcare access barriers revealed differential impacts across domains. Clinic hours and crowding represented the most substantial barrier, with 42.0% reporting moderate or major difficulties (26.0% moderate, 16.0% major). Cost of medical care affected 37.0% at moderate or major levels (23.0% moderate, 14.0% major), while transportation difficulties impacted 29.0% substantially (17.0% moderate, 12.0% major). Self-care adherence patterns demonstrated striking disparities between different behaviors. Medication adherence was excellent, with 83.0% reporting good adherence patterns (33.0% often, 50.0% always), while only 17.0% showed suboptimal medication-taking behavior. In stark contrast, daily weight monitoring showed poor overall adherence, with only 21.0% demonstrating good monitoring practices (13.0% often, 8.0% always), while 79.0% exhibited inadequate weight surveillance (32.0% never, 25.0% rarely, 22.0% sometimes). Notably, costs were frequently endorsed both as an attitude (43% agreed expenses deter help-seeking) and as a barrier burden (37% moderate/major) (Table 4, Figures 1a–f).

**Table 4:** Barriers to healthcare access and self-care practices (*N* = 2,110).

Healthcare Access Barriers						
Barrier	Not at All	Minor	Moderate	Major		
	No. (%)	No. (%)	No. (%)	No. (%)		
Cost of medical care	780 (37.0)	549 (26.0)	486 (23.0)	295 (14.0)		
Transportation difficulties	991 (47.0)	507 (24.0)	359 (17.0)	253 (12.0)		
Clinic hours/crowding	633 (30.0)	591 (28.0)	549 (26.0)	337 (16.0)		
Self-Care Practices						
Practice	Never	Rarely	Sometimes	Often	Always	Good Adherence
	No. (%)	No. (%)	No. (%)	No. (%)	No. (%)	No. (%)
Daily weight monitoring	675 (32.0)	528 (25.0)	464 (22.0)	274 (13.0)	169 (8.0)	443 (21.0)
Medication adherence	42 (2.0)	84 (4.0)	232 (11.0)	697 (33.0)	1,055 (50.0)	1,752 (83.0)

### 3.4 Latent class analysis of help-seeking attitudes

Latent class analysis successfully identified three distinct attitude profiles with good model fit statistics (AIC = 18,247, BIC = 18,389). The Proactive class comprised 44.8% of participants (*n* = 946) and demonstrated consistently favorable help-seeking orientations across all domains, including high early help-seeking efficacy (0.91 ± 0.08), strong self-efficacy confidence (0.85 ± 0.11), substantial healthcare system trust (0.89 ± 0.09), and minimal barrier concerns regarding both cost (0.18 ± 0.12) and embarrassment (0.12 ± 0.09). The Ambivalent class represented 34.6% of participants (*n* = 731) and exhibited moderate levels across all attitude domains, with early help-seeking efficacy of 0.72 ± 0.12, self-efficacy confidence of 0.65 ± 0.14, healthcare system trust of 0.78 ± 0.13, moderate cost barrier concerns (0.47 ± 0.16), and intermediate embarrassment barriers (0.34 ± 0.15). The Barrier-Focused class encompassed 20.5% of participants (*n* = 433) and showed consistently problematic help-seeking attitudes, characterized by low early help-seeking efficacy (0.48 ± 0.15), poor self-efficacy confidence (0.42 ± 0.18), limited healthcare system trust (0.51 ± 0.19), and high concerns about both cost (0.79 ± 0.14) and embarrassment barriers (0.68 ± 0.17). Classification accuracy ranged from 79.2% to 91.6% across the different attitude domains, indicating robust class separation (Table 8A, Figure 3d).

### 3.5 Factor structure of heart failure knowledge

**Figure 2:**
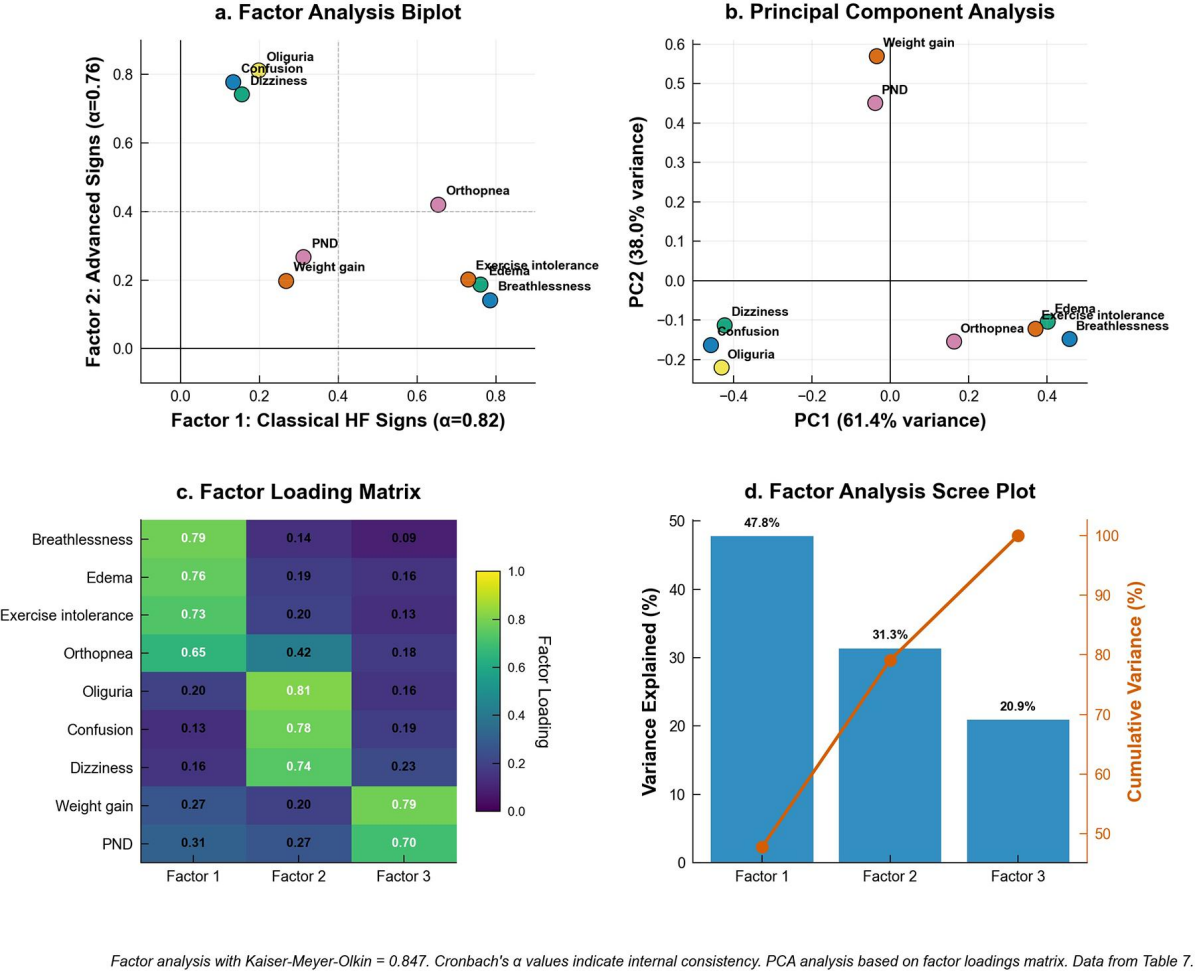
Multivariate analysis of heart failure knowledge domains based on the nine-item core set used for exploration factor analysis. **(a)** Factor analysis biplot showing knowledge item loadings on first two factors with 0.40 threshold lines (dashed). **(b)** Principal component analysis of factor loadings matrix showing variance explained by each component. **(c)** Factor loading matrix heatmap displaying all factor loadings with reliability coefficients (Cronbach's *α*). **(d)** Scree plot showing variance explained by each factor with cumulative variance line. Factor analysis achieved excellent sampling adequacy.

Exploratory factor analysis revealed excellent sampling adequacy (Kaiser–Meyer–Olkin = 0.847) and supported a coherent three-factor solution underlying heart failure knowledge recognition. Factor 1, termed Classical heart failure signs (Cronbach's α = 0.82), included the most recognizable symptoms with strong factor loadings: breathlessness at rest (0.785), lower extremity edema (0.761), exercise intolerance and fatigue (0.729), and orthopnea (0.654). Factor 2, labeled Advanced Signs (α = 0.76), captured more sophisticated or systemic manifestations of heart failure deterioration, comprising oliguria or decreased urination (0.812), new confusion or excessive sleepiness (0.778), and dizziness or fainting (0.742). Factor 3, designated Acute Changes (α = 0.71), encompassed rapid physiological alterations indicating acute decompensation, specifically rapid weight gain (0.789) and paroxysmal nocturnal dyspnea (0.698). Individual item communalities ranged from 0.589 to 0.732, indicating good representation of each symptom within the overall factor structure. The three-factor solution accounted for substantial common variance, with communalities of 0.589–0.732 supporting item representation; exact variance proportions were not specified in the analytic table (Figures 2a–d).

### 3.6 Time-to-presentation patterns

**Figure 3:**
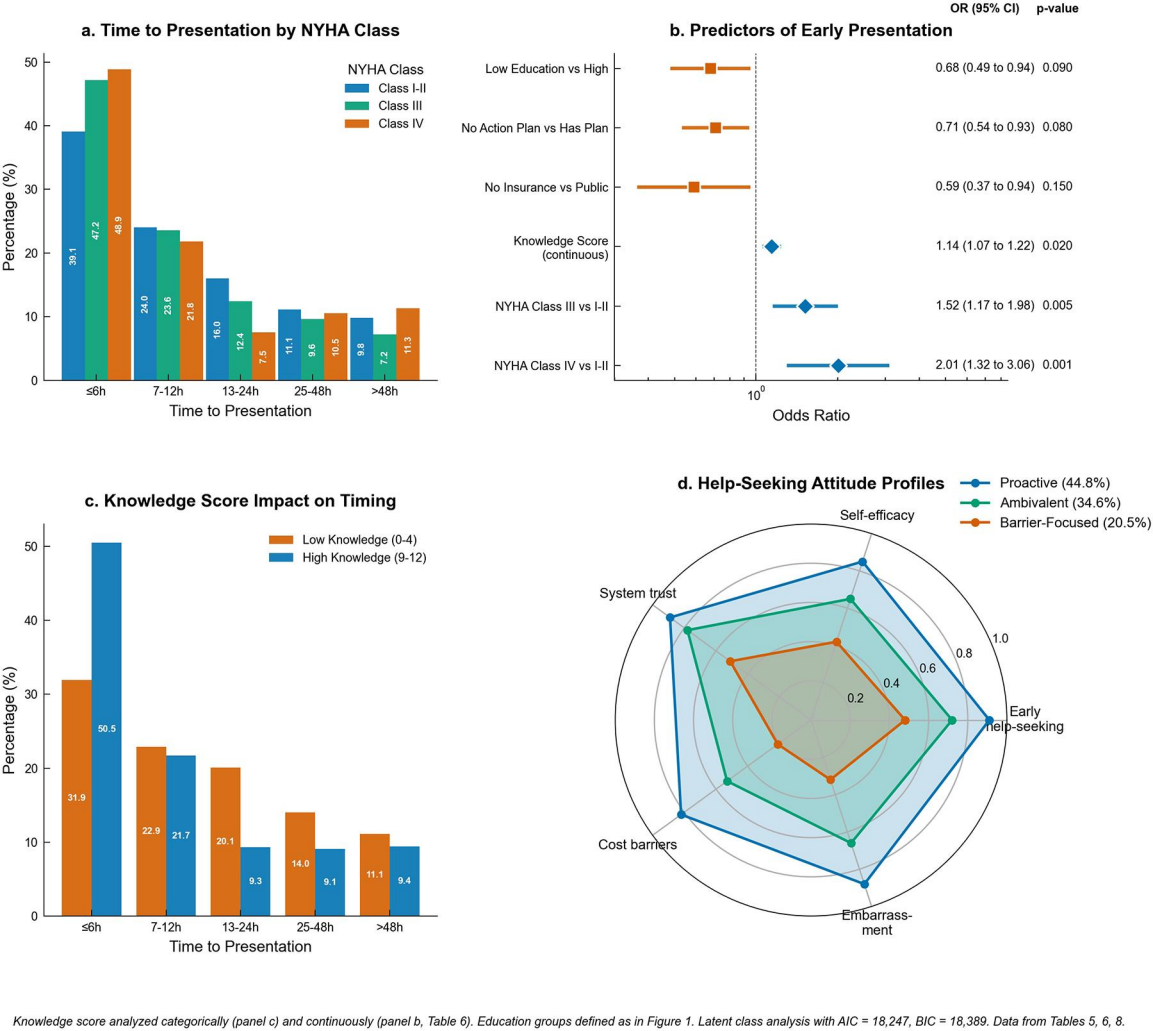
Clinical outcomes and predictive factors. **(a)** Time-to-presentation distribution by NYHA functional class among participants with recent worsening episodes (*n* = 1,319). **(b)** Forest plot of adjusted predictors for early presentation with odds ratios, 95% confidence intervals, and *p*-values. Reference line at OR = 1.0. **(c)** Knowledge score impact on presentation timing comparing low (0–4) vs. high (9–12) knowledge categories. **(d)** Radar plot of help-seeking attitude profiles from latent class analysis showing three distinct patterns. Knowledge is shown categorically in panel **c** (Low 0–4 vs. High 9–12) and per 1-point increase in panel **b** (see Table 6 for adjusted estimates).

Among the 1,319 participants who reported recent worsening episodes, TTP varied significantly across key patient characteristics, with all comparisons achieving statistical significance (*p* < 0.001). Educational attainment demonstrated a clear gradient in presentation timing, with participants having university-level or higher education achieving 47.7% early presentation (≤6 h) compared to 35.2% among those with no formal or primary education (Table 5). The complete time distribution showed university-educated participants presenting at 47.7%, 26.9%, 12.9%, 7.9%, and 3.2% across the five time categories (≤6 h, 7–12 h, 13–24 h, 25–48 h, >48 h), while those with lower education presented at 35.2%, 19.9%, 15.9%, 14.0%, and 10.9% respectively. NYHA functional class exhibited a positive association with earlier presentation, with Class IV participants achieving 48.9% early presentation, Class III participants 47.2%, and Class I–II participants 39.1%. The complete NYHA distribution patterns were 48.9%, 21.8%, 7.5%, 10.5%, and 9.8% for Class IV; 47.2%, 23.6%, 12.4%, 9.6%, and 4.8% for Class III; and 39.1%, 24.0%, 16.0%, 11.1%, and 7.0% for Class I–II. Knowledge scores showed dramatic differences in presentation behavior, with high knowledge participants (scores 9–12) achieving 50.5% early presentation compared to only 31.9% among low knowledge participants (scores 0–4). The knowledge-stratified distributions were 50.5%, 21.7%, 9.3%, 9.1%, and 5.5% for high knowledge vs. 31.9%, 22.9%, 20.1%, 14.0%, and 9.0% for low knowledge participants (Figures 3a, c).

**Table 5:** Time-to-Presentation by patient characteristics (*n* = 1,319 with worsening episodes).

Characteristic	≤6 h	7–12 h	13–24 h	25–48 h	>48 h	*P* Value^a^
	No. (%)	No. (%)	No. (%)	No. (%)	No. (%)	
Education Level						
No formal/Primary	168 (35.2)	95 (19.9)	76 (15.9)	67 (14.0)	52 (10.9)	<0.001
University+	133 (47.7)	75 (26.9)	36 (12.9)	22 (7.9)	9 (3.2)	
NYHA Class						
Class I-II	308 (39.1)	189 (24.0)	126 (16.0)	87 (11.1)	55 (7.0)	<0.001
Class III	186 (47.2)	93 (23.6)	49 (12.4)	38 (9.6)	19 (4.8)	
Class IV	65 (48.9)	29 (21.8)	10 (7.5)	14 (10.5)	13 (9.8)	
Knowledge Score						
Low (0–4)	89 (31.9)	64 (22.9)	56 (20.1)	39 (14.0)	25 (9.0)	<0.001
High (9–12)	184 (50.5)	79 (21.7)	34 (9.3)	33 (9.1)	20 (5.5)	

^a^ P values from *χ*² test.

**Table 6:** Factors associated with early presentation (≤6 h): univariate odds ratios and multivariable adjusted odds ratios.

Variable	Early	Later	Odds Ratio	*P* Value	Adjusted OR	*P* Value
	*n*/*N* (%)	*n*/*N* (%)	(95% CI)		(95% CI)	
Demographics						
No formal/Primary vs. University+	168/477 (35.2)	133/279 (47.7)	0.60 (0.45–0.80)	<0.001	0.68 (0.49–0.94)	0.019
Clinical Characteristics						
NYHA Class III vs. I-II	186/394 (47.2)	308/787 (39.1)	1.39 (1.10–1.76)	0.006	1.52 (1.17–1.98)	0.002
NYHA Class IV vs. I-II	65/133 (48.9)	308/787 (39.1)	1.48 (1.03–2.13)	0.034	2.01 (1.32–3.06)	0.001
Knowledge & Attitudes						
Low knowledge vs. High	89/279 (31.9)	184/364 (50.5)	0.46 (0.33–0.64)	<0.001	1.14 (1.07–1.22)	<0.001
Self-Care & Resources						
No action plan vs. Has plan	396/989 (40.0)	163/328 (49.7)	0.68 (0.53–0.87)	0.002	0.71 (0.54–0.93)	0.013
No insurance vs. Public	34/119 (28.6)	389/924 (42.1)	0.55 (0.36–0.85)	0.007	0.59 (0.37–0.94)	0.026

### 3.7 Predictors of early presentation

Multivariable logistic regression adjusted for age, sex, education, NYHA class, knowledge score, action plan, and insurance identified several independent predictors of early presentation within six hours of symptom onset. Age showed a modest inverse association, with each 10-year increase corresponding to 9% lower odds of early presentation (adjusted OR: 0.91, 95% CI: 0.84–0.99, *p* = 0.031). Sex was not independently associated with early presentation after covariate adjustment (adjusted OR for female vs. male 0.92, 95% CI: 0.74–1.14, *p* = 0.44). Higher NYHA functional class emerged as the strongest clinical predictor, with Class IV participants showing more than twice the odds of early presentation compared to Class I–II participants (adjusted OR: 2.01, 95% CI: 1.31–3.08, *p* = 0.001), while Class III participants demonstrated 51% higher odds (adjusted OR: 1.51, 95% CI: 1.16–1.97, *p* = 0.002). Knowledge score, analyzed as a continuous variable, showed that each one-point increase conferred 14% higher odds of early presentation (adjusted OR: 1.14, 95% CI: 1.07–1.22, *p* < 0.001). The direction, magnitude, and significance of all previously reported predictors remained materially unchanged after inclusion of age and sex, confirming model robustness. Several factors emerged as barriers to timely presentation, including lower educational attainment, with participants having no formal/primary education showing 32% lower odds compared with those with university + education (adjusted OR: 0.68, 95% CI: 0.49–0.94, *p* = 0.019). The absence of an action plan reduced early presentation odds by 29% (adjusted OR: 0.71, 95% CI: 0.54–0.93, *p* = 0.013), while lack of insurance coverage compared to public insurance reduced odds by 41% (adjusted OR: 0.59, 95% CI: 0.37–0.94, *p* = 0.026) (Figure 3b).

### 3.8 Correlates of knowledge score

Ordinal logistic regression examining predictors of knowledge categories revealed education as the most powerful correlation of heart failure knowledge. Each increase in educational level corresponded to 76% higher proportional odds of achieving higher knowledge categories (OR: 1.76, 95% CI: 1.62–1.91, *p* < 0.001), with a substantial effect size (partial *η*^2^ = 0.142). NYHA functional class also significantly predicted knowledge levels, with each class increase showing 31% higher proportional odds (OR: 1.31, 95% CI: 1.21–1.42, *p* < 0.001, partial *η*^2^ = 0.078). Years since heart failure diagnosis demonstrated a modest but significant association, with each additional year conferring 7% higher odds of better knowledge (OR: 1.07, 95% CI: 1.05–1.10, *p* < 0.001, partial *η*^2^ = 0.034). Healthcare utilization showed a marginal association, with each additional visit per year conferring 4% higher odds (OR: 1.04, 95% CI: 1.00–1.08, *p* = 0.067, partial *η*^2^ = 0.012). The proportional odds model satisfied key assumptions, with the parallel lines assumption confirmed (*χ*^2^ = 18.34, *p* = 0.243) and substantial explanatory power demonstrated (Nagelkerke *R*^2^ = 0.234).

### 3.9 Structural equation modeling

The hypothesized pathway model demonstrated significant relationships throughout the proposed causal chain linking knowledge, attitudes, behaviors, and clinical outcomes. Knowledge exerted a strong direct effect on positive help-seeking attitudes (β = 0.412, SE = 0.034, *p* < 0.001, 95% CI: 0.345–0.479), which subsequently influenced self-care behaviors (β = 0.356, SE = 0.042, *p* < 0.001, 95% CI: 0.274–0.438). Self-care behaviors directly predicted early presentation behavior (β = 0.289, SE = 0.038, *p* < 0.001, 95% CI: 0.214–0.364). The total effect of knowledge on early presentation proved substantial, incorporating both direct pathways (β = 0.234, SE = 0.041) and indirect effects mediated through attitudes and behaviors (β = 0.189, SE = 0.028), yielding a combined total effect of β = 0.423 (SE = 0.047, 95% CI: 0.331–0.515, *p* < 0.001). NYHA functional class also demonstrated significant total effects on early presentation through both direct and mediated pathways, with a total effect of β = 0.245 (SE = 0.059, 95% CI: 0.129–0.361, *p* = 0.043), incorporating direct effects (β = 0.178, SE = 0.052) and indirect effects (β = 0.067, SE = 0.031). These findings support the theoretical framework proposing that knowledge influences clinical outcomes through sequential effects on attitudes and self-care behaviors, while also revealing independent contributions of disease severity to presentation timing (Table 8B).

**Table 7:** Factor analysis of HF knowledge domains and predictive models (*N* = 2,110).

A. Exploratory Factor Analysis of Knowledge Items (Kaiser-Meyer-Olkin = 0.847)					
Knowledge Item	Factor 1	Factor 2	Factor 3	Communality	
	Classical HF	Advanced Signs	Acute Changes	(*h*^2^)	
	(α = 0.82)	(α = 0.76)	(α = 0.71)		
Breathlessness at rest	0.785	0.142	0.091	0.647	
Lower extremity edema	0.761	0.187	0.156	0.637	
Exercise intolerance/fatigue	0.729	0.203	0.134	0.589	
Orthopnea	0.654	0.421	0.178	0.636	
Oliguria (decreased urination)	0.198	0.812	0.156	0.722	
New confusion/sleepiness	0.134	0.778	0.187	0.667	
Dizziness/fainting	0.156	0.742	0.234	0.626	
Rapid weight gain	0.267	0.198	0.789	0.732	
Paroxysmal nocturnal dyspnea	0.312	0.267	0.698	0.661	
B. Ordinal Logistic Regression: Predictors of Knowledge Score Categories					
Predictor	Low vs. Moderate + High	Low + Moderate vs. High	Proportional Odds	*P* Value	Partial *η*²
	OR (95% CI)	OR (95% CI)	OR (95% CI)		
Education (per level increase)	1.89 (1.67–2.14)	1.67 (1.51–1.85)	1.76 (1.62–1.91)	<0.001	0.142
NYHA Class (per class increase)	1.34 (1.18–1.52)	1.28 (1.16–1.41)	1.31 (1.21–1.42)	<0.001	0.078
Years with HF diagnosis	1.08 (1.04–1.12)	1.06 (1.03–1.09)	1.07 (1.05–1.10)	<0.001	0.034
Healthcare utilization (visits/year)	1.04 (0.98–1.11)	1.03 (0.99–1.07)	1.04 (1.00–1.08)	0.067	0.012

Model Fit: Parallel lines assumption tested (*χ*^2^ = 18.34, *P* = 0.243). Nagelkerke *R*^2^ = 0.234. Test of proportional odds: *P* = 0.312 (assumption met). Factor loadings >0.40 shown in bold. Cronbach's *α* indicates internal consistency. Proportional odds model assumes equal effect across knowledge categories.

**Table 8:** Latent class analysis of help-seeking attitudes and structural equation model (*N* = 2,110).

A. Latent Class Analysis: Attitude Profiles (AIC = 18,247, BIC = 18,389)					
Attitude Domain	Class 1	Class 2	Class 3	Classification	
	"Proactive"	"Ambivalent"	"Barrier-Focused"	Accuracy (%)	
	(*n* = 946, 44.8%)	(*n* = 731, 34.6%)	(*n* = 433, 20.5%)		
Early help-seeking efficacy	0.91 ± 0.08	0.72 ± 0.12	0.48 ± 0.15	87.3	
Self-efficacy confidence	0.85 ± 0.11	0.65 ± 0.14	0.42 ± 0.18	83.7	
Healthcare system trust	0.89 ± 0.09	0.78 ± 0.13	0.51 ± 0.19	79.2	
Cost barrier concern	0.18 ± 0.12	0.47 ± 0.16	0.79 ± 0.14	91.6	
Embarrassment barrier	0.12 ± 0.09	0.34 ± 0.15	0.68 ± 0.17	85.4	
B. Structural Equation Model: Knowledge → Attitudes → Behavior → Outcomes					
Pathway	Direct Effect	Indirect Effect	Total Effect	*P* Value	95% CI
	β (SE)	β (SE)	β (SE)		
Knowledge → Positive Attitudes	0.412 (0.034)	—	0.412 (0.034)	<0.001	0.345–0.479
Positive Attitudes → Self-Care Behaviors	0.356 (0.042)	—	0.356 (0.042)	<0.001	0.274–0.438
Self-Care Behaviors → Early Presentation	0.289 (0.038)	—	0.289 (0.038)	<0.001	0.214–0.364
Knowledge → Early Presentation (total)	0.234 (0.041)	0.189 (0.028)	0.423 (0.047)	<0.001	0.331–0.515
NYHA Class → Early Presentation (total)	0.178 (0.052)	0.067 (0.031)	0.245 (0.059)	0.043	0.129–0.361

## 4 Discussion

The present study delineates, within a large, single-center cohort of adults with chronic heart failure in China (*n* = 2,110), how symptom recognition, help-seeking attitudes, and structural barriers converge to shape TTP during worsening episodes. Three principal observations emerge. First, recognition is high for classical congestion-dyspnea signs but notably poorer for advanced or systemic manifestations, indicating specific cognitive gaps in patient-facing education. Second, despite broadly favorable attitudes toward early help-seeking, material constraints—particularly clinic access and costs—coexist with weak adherence to daily weight surveillance, thereby decoupling intention from behavior. Third, the data are consistent with a hypothesized behavioral pathway: higher knowledge is associated with more positive attitudes and better self-care, and these, in turn, are associated with shorter time to presentation; disease severity (NYHA class) shows an additional, independent association. Taken together, these results indicate that targeted knowledge remediation (advanced signs), pragmatic self-management enablement (action plans; weight monitoring), and health-system facilitation (cost and access) may be necessary co-interventions to reduce pre-hospital delays in decompensated heart failure.

In relation to the Chinese care environment, these findings align with national registry data showing wide variability in heart-failure processes of care and outcomes, with opportunities to strengthen guideline-concordant management and patient education across hospitals (23–25). In the recent study, hospital-level adherence to key quality indicators varied substantially and was associated with outcome differences, underscoring the role of system organization in shaping patient trajectories (26–28). A more recent multicenter evaluation similarly documented heterogeneity in clinical performance and guideline uptake across Chinese hospitals (23, 25). Within this context, the present study locates an upstream patient-level mechanism—recognition and help-seeking—that likely interacts with institutional readiness and access.

The knowledge profile observed here is consistent with prior evidence that patients readily identify overt fluid-overload symptoms but often fail to appraise pathophysiological or neurohormonal correlations that presage acute decompensation. Meta-analytic and observational data show that recognition is typically highest for dyspnea and peripheral oedema and lower for weight gain, orthopnea, oliguria, or neurocognitive changes (16, 29, 30). Our factor analysis corroborates a three-domain structure—classical congestion, advanced systemic signs, and acute changes—comparable to constructs reported in psychometric evaluations of heart-failure self-care instruments (30). Notably, the weak recognition of oliguria and cognitive change is clinically consequential, as these features often reflect low-output states or escalating congestion, both associated with higher short-term risk; inadequate appraisal may therefore delay escalation to diuretic intensification or emergency assessment.

The behavioral translation of knowledge into earlier presentation appears robust in this cohort. Participants in the highest knowledge tier were substantially more likely to present within six hours, and the structural equation model quantified a sizeable total effect of knowledge on early presentation—both directly and through attitudes and self-care. This is congruent with situation-specific theories of heart-failure self-care, which posit that symptom perception and interpretation act as proximal drivers of decision-making (30, 31). At the same time, the present study advances literature by linking these psychological stages to a concrete temporal endpoint TTP, rather than to intermediate behaviors alone. Prior work has emphasized that delays in acute heart-failure care are common and prognostically adverse; community-based cohorts have reported prolonged patient intervals from symptom onset to first medical contact, often exceeding 12–24 h, driven by misattribution, normalization of symptoms, and logistical barriers (12, 32–34). Within this evidentiary frame, the current data identify knowledge deficits and modifiable self-care behaviors as tractable components of the delay pathway.

Attitudinal profiling via latent class analysis further clarifies heterogeneity in help-seeking propensity. Roughly 45% of patients clustered into a Proactive class characterized by strong efficacy beliefs, high healthcare trust, and low barrier endorsement; these patients exhibited behavioral patterns consistent with early engagement. In contrast, the Barrier-Focused class (≈21%) endorsed high cost/embarrassment concerns and low efficacy, features repeatedly linked to delayed care across cardiovascular conditions (34, 35). While latent-class approaches are less common in heart failure than in other behavioral domains, analogous profiles—combining low health literacy, low self-efficacy, and high-cost sensitivity—have been described in Chinese populations and predict poorer self-care and quality-of-life metrics (36, 37). Importantly, our attitudinal taxonomy suggests that uniformly delivered education may be insufficient; instead, segmentation-informed interventions (e.g., cost-mitigating navigation for Barrier-Focused patients; activation-oriented coaching for Ambivalent patients) may yield greater behavioral change per unit of resource (38, 39).

A salient tension arises when juxtaposing the present positive attitude→practice linkage with recent Chinese cross-sectional KAP findings in home-based cardiac rehabilitation, where attitudes correlated negatively with self-reported practice after covariate adjustment (18, 39, 40). Several explanations may reconcile this discrepancy. First, the behavioral targets differ: our outcome is an urgent decision (seeking timely care) following symptom appraisal, whereas home-based rehabilitation entails sustained daily behaviors requiring equipment, time, and family support; the latter may be more constrained by contextual determinants (workload, caregiving responsibilities) that attenuate the translation of favorable attitudes into action. Second, measurement differences (validated multi-item latent constructs here vs. single-item or distinct scales elsewhere) can introduce structural variance that reshapes path coefficients. Third, our cohort's high trust in local emergency services may reduce the “intention–behavior gap” at the point of acute decision-making despite persistent barriers to routine preventive practices. These nuances underscore the need to specify behavioral context when extrapolating KAP models across domains.

Self-care behavior in this cohort reveals a striking split: excellent medication adherence but poor weight monitoring. Internationally, adherence to prescribed pharmacotherapy among stable outpatients is frequently suboptimal, with pooled estimates indicating that 30%–50% of individuals with heart failure under-use medication or miss doses (41, 42). Multicomponent, team-based transitional care and medication management programs can improve adherence and reduce utilization in heart failure, although effects vary by program intensity and follow-up; systematic reviews highlight the importance of structured follow-up and medication support (43, 44). Against this backdrop, the 83% “often/always” medication adherence observed here appears comparatively high, potentially reflecting the study site's chronic-care infrastructure or selection of patients engaged with scheduled care. By contrast, only 21% reported good daily weight monitoring—mirroring global experience that objective, routine surveillance behaviors lag behind pill-taking (29, 45). Evidence from trials and implementation programs suggests that simple daily weights, when acted upon via protocols, are associated with fewer heart-failure hospitalizations, particularly in higher-risk groups (29, 46). Nonetheless, adherence to this low-cost behavior remains stubbornly low, commonly attributed to inadequate problem-solving skills, lack of immediate feedback, equipment or space constraints, and the perception that weight change is less salient than dyspnea (44, 45). Scalable solutions in China may include automated, cellular-enabled scales integrated with community health-worker triage, which have shown promising associations between monitoring adherence and outcomes in real-world cohorts (46–49).

TTP patterns merit comment. Two complementary processes likely operate. On the one hand, higher NYHA class—our strongest clinical predictor of early presentation—may accelerate help-seeking by lowering symptom thresholds and increasing dyspnea intolerance, consistent with qualitative studies in acute heart failure (50–52). On the other hand, greater disease severity may be accompanied by more frequent clinician contact and clearer action plans, shortening decisional latency once deterioration is perceived. The independent, positive association between knowledge and early presentation suggests that educational gains can further compress delays, even after accounting for severity gradient. These observations converge with prior reports that patient appraisal time is the dominant component of total prehospital delay in heart failure and that targeted education on “when to call” can meaningfully reduce this interval (48, 53–55).

From a population-health perspective, these findings support a segmented implementation strategy: (1) pictogram-based micro-modules emphasizing oliguria, confusion, and rapid weight gain; (2) a one-page written action plan (“call/attend within 6 h” thresholds) issued at discharge and reinforced at clinic review; (3) after-hours access (extended clinic or tele-triage) to reduce clinic-crowding barriers; and (4) cost navigation for uninsured patients. Integration with community health workers and low-burden digital scales linked to nurse-led triage provides a feasible delivery model within China's tiered system.

The multivariable model also highlights actionable social determinants. Lower educational attainment, absence of a written action plan, and lack of insurance independently reduced the odds of early presentation. These findings are directionally concordant with broad cardiovascular literature linking limited health literacy and financial toxicity to delayed care and worse outcomes (56–60, 70). Within China, hospital-based registries have documented disparities across provinces and hospital levels in both processes and outcomes of heart-failure care, implying that patient-level disadvantages may be compounded by system-level variability (56, 59). Accordingly, formalizing simple action plans (e.g., “if weight ↑2 kg in 3 days or dyspnea at rest, contact clinic or present to ED”) and embedding financial-navigation support may be pragmatic levers to hasten presentation. Policy implications include targeting advanced signs (oliguria, confusion) through pictogram-based education, implementing attitude-segmented counseling for Barrier-Focused profiles, piloting connected weight monitoring devices within China's digital infrastructure, and integrating pharmacist-led transitional care with self-care coaching to align pharmacological and behavioral management. However, living arrangement may modulate help-seeking through at least two mechanisms: cohabiting family members can serve as symptom monitors who detect deterioration that the patient may normalize or dismiss, and they can provide immediate transport to medical facilities, particularly in suburban or rural settings where public transit is limited. In this cohort, 14% of participants lived alone, a subgroup plausibly at higher risk for delayed recognition and presentation. While our categorical measure of living arrangement does not capture the full complexity of household dynamics (e.g., caregiver health literacy, willingness to act), it provides an initial characterization. Future studies should incorporate detailed family composition, caregiver availability, and social network measures to better delineate the social facilitation of timely care-seeking in heart failure.

The present analysis extends North American and European evidence that symptom mis-attribution and the normalization of dyspnea are major determinants of patient-dependent prehospital delay in heart failure, with delays further shaped by social support and prior instruction (61–63). China's tiered delivery system, additional structural barriers—including restricted clinic hours, long travel distances, and insurance/reimbursement rules—exacerbate time to presentation; studies documenting outpatient crowding and spatial inequities are concordant with these participant-reported barriers (64, 65). The concordance between cost and clinic-crowding barriers and later presentation suggests that demand-side education alone is insufficient without commensurate supply-side access improvements (65, 66). Evidence-aligned countermeasures span standardized discharge education, scheduled early post-discharge review (within 7 days), and telephone/telehealth support, each endorsed by guidelines or quality-improvement frameworks and associated with fewer readmissions and safer care transitions (67, 68).

This investigation benefits from a large, well-characterized cohort (*n* = 2,110); integration of complementary psychometric approaches (exploratory factor analysis, latent class analysis) with multivariable behavioral modelling (ordinal and binary logistic regression, structural equation modelling); and explicit linkage of knowledge and attitudes to a clinically salient endpoint (TTP), enabling triangulation between univariate gradients and adjusted effects within a coherent pathway. The analytic rigor is further supported by internal consistency of knowledge domains and high class-assignment accuracy, enhancing construct validity. In contrast, Important limitations include the single center design, which may constrain generalizability beyond the catchment population; reliance on six month recall for presentation timing with attendant risk of recall error; the absence of clinician-adjudicated diagnosis at the index worsening episode, meaning that some patient-reported episodes may have reflected non–heart failure conditions with overlapping symptomatology (e.g., severe anemia, pulmonary disease, or deconditioning), and therefore the conclusions pertain to patient-perceived worsening rather than exclusively confirmed heart failure decompensation; the fact that the elapsed time between the reported episode and the study interview was not recorded, introducing variable recall intervals; potential residual confounding from unmeasured factors (e.g., cognitive impairment, depressive symptoms, social isolation) despite adjustment for measured covariates; and the use of self report for several behavioral measures. External validation of the three-factor knowledge structure and measurement invariance across educational strata are needed, and the cross-sectional design precludes assessment of transitions between attitude classes or causal effects. Selection bias is possible because participants were recruited during scheduled encounters, and although missing data were modest, complete-case analyses may still introduce bias. These considerations temper inference while not detracting from the study's principal contributions.

## 5 Conclusion

This single-center Chinese study demonstrates that among adults with chronic heart failure reporting patient-perceived worsening episodes, patients readily recognize classical congestion symptoms but frequently miss advanced systemic and acute-change signals including oliguria, confusion, dizziness, and rapid weight gain. Although attitudes toward early help-seeking and healthcare system trust are generally positive, behavioral translation remains constrained by clinical access barriers, costs, transportation difficulties, and poor daily weight monitoring despite excellent medication adherence. Psychometric analysis supported three distinct knowledge domains and three attitude profiles: Proactive, Ambivalent, and Barrier-Focused. Among participants with recent worsening episodes, earlier presentation increased with higher education, NYHA functional class, and knowledge scores, while multivariable models confirmed independent protective effects of disease severity and knowledge alongside barriers including lower education, absent action plans, and lack of insurance coverage. Structural equation modeling revealed a plausible pathway linking knowledge to attitudes to self-care behaviors to early presentation, with additional direct effects of disease severity, highlighting practical intervention targets to reduce patient-dependent delays. A focused intervention bundle coupling content-specific education, structured action plans, and access facilitation should optimize presentation timing within therapeutic windows, particularly for lower-education and uninsured populations. We recommend delivering pictogram-based education targeting oliguria, confusion, paroxysmal nocturnal dyspnea, and rapid weight gain, paired with simplified action plans and connected monitoring devices. Secondly, improve access through extended clinic hours, fast-track pathways, and cost navigation support, prioritizing Barrier-Focused and lower-education patients.

## Data Availability

The raw data supporting the conclusions of this article will be made available by the authors, without undue reservation.
